# *Enterocloster alcoholdehydrogenati* sp. nov., a Novel Bacterial Species Isolated from the Feces of a Patient with Alcoholism

**DOI:** 10.1007/s00284-023-03285-1

**Published:** 2023-04-19

**Authors:** Daiki Oikawa, Kohei Fukui, Yuichi Aoki, Toshiyuki Waki, Seiji Takahashi, Takefumi Shimoyama, Toru Nakayama

**Affiliations:** 1 Department of Biomolecular Engineering, Graduate School of Engineering, Tohoku University, Aoba 6-6-11, Aramaki, Aoba-Ku, Sendai, Miyagi 980-8579 Japan; 2 Division of Integrated Life Science, Graduate School of Biostudies, Kyoto University, Oiwake-Cho, Kitashirakawa, Sakyo-Ku, Kyoto, 606-8502 Japan; 3 Department of Integrative Genomics, Tohoku Medical Megabank Organization, Seiryo 2-1, Sendai, Miyagi 980-8573 Japan; 4 Graduate School of Information Sciences, Tohoku University, Aoba 6-3-09, Aramaki, Aoba-Ku, Sendai, Miyagi 980-8679 Japan

## Abstract

Strain C5-48^T^, an anaerobic intestinal bacterium that potentially accumulates acetaldehyde at levels exceeding its minimum mutagenic concentration (50 µM) in the colon and rectum, was isolated from the feces of a patient with alcoholism. The 16S rRNA gene sequence of strain C5-48^T^ showed high similarity to the corresponding sequences of *Lachnoclostridium edouardi* Marseille-P3397^T^ (95.7%) and *Clostridium fessum* SNUG30386^T^ (94.7%). However, phylogenetic analysis using the sequences of the 16S rRNA, *rpoB*, and *hsp60* genes and whole-genome analysis strongly suggested that C5-48^T^ should be included in the genus *Enterocloster*. The novelty of strain C5-48^T^ was further confirmed by comprehensive average nucleotide identity (ANI) calculations based on its whole-genome sequence, which showed appreciable ANI values with known *Enterocloster* species (*e.g*., 74.3% and 73.4% with *Enterocloster bolteae* WAL 16351^T^ and *Enterocloster clostridioformis* ATCC 25537^T^, respectively). The temperature range for growth of strain C5-48^T^ was 15–37 °C with an optimum of 37 °C. The pH range for growth was 5.5–10.5 with an optimum of 7.5. The major constituents of the cell membrane lipids of strain C5-48^T^ were 16:0, 14:0, and 18:1 *ω*7*c* dimethyl acetal fatty acids. On the basis of the genotypic and phenotypic properties, *Enterocloster alcoholdehydrogenati* sp. nov. is proposed, with the type strain C5-48^T^ (= JCM 33305^T^ = DSM 109474^T^).

**Supplementary Information:**

The online version contains supplementary material available at 10.1007/s00284-023-03285-1.

## Introduction

Chronic consumption of excess alcohol induces colonic lesions, colonic inflammation, and oxidative stress in the gut, increasing the risk of colorectal cancer [1, 2]. Acetaldehyde (AcH) is produced during ethanol metabolism mediated by intestinal bacteria and colorectal mucosal cells. The minimum mutagenic concentration (MMC) of acetaldehyde has been estimated as 50 µM [3]. AcH associated with alcohol consumption is a potential risk factor for alcohol-related digestive tract cancers such as those of the esophagus, colon, and rectum. In 2010, the World Health Organization’s International Agency for Research on Cancer concluded that alcohol-consumption-associated AcH is carcinogenic to humans [4]. Intestinal bacteria have been implicated in the accumulation of AcH in the colon and rectum after drinking alcohol [5, 6], although the ecophysiological details remain to be clarified. Because colorectal cancer develops from mucosal cells, the populations of AcH-accumulating bacteria inhabiting the colorectal mucosal surface may play a key role in alcohol-related colorectal cancer pathogenesis [5, 6].

Tsuruya et al*.* comprehensively examined the ability of bacterial isolates from the feces of patients with alcoholism to produce and decompose AcH [6, 7]. They identified various microorganisms that potentially accumulate AcH at levels higher than the MMC in the colon and rectum [6]. These microorganisms were collectively referred to as potential AcH accumulators [6, 7], and included strain C5-48^T^. Phylogenetic analysis based on the 16S rRNA gene sequence suggested that strain C5-48^T^ was related to the genus *Clostridium* [6], in particular, *C. sphenoides* and *C. clostridioforme* (see below). Recently, *C. sphenoides*, *C. clostridioforme*, and other related taxa were collectively reclassified as strains of *Lacrimispora* gen. nov. and *Enterocloster* gen. nov. [8]. Thus, we have carefully compared the taxonomic characteristics of C5-48^T^ with known species of *Lacrimispora*, *Enterocloster*, *Clostridium*, and related genera, and proposed that C5-48^T^ is a new species of the genus *Enterocloster*, for which the name *Enterocloster alcoholdehydrogenati* sp. nov. is proposed.

## Materials and Methods

### Isolation and Culture Conditions of Strain C5-48^T^

Strain C5-48^T^ was isolated from the feces of a Japanese male patient with alcoholism (AL05) [7] at Kurihama Medical and Addiction Center, Kanagawa, Japan, as described previously [6, 7]. At the time of fecal sample collection, this patient was 60 years old and had been a habitual drinker for 40 years, with an estimated average intake of 88 g of pure alcohol per day, and continued drinking until 7 days before fecal sample collection. He had also been a habitual smoker for 40 years, smoking 15 cigarettes per day. For other clinical details of AL05, see reference [7].

Cells of strain C5-48^T^ were grown at 37 °C overnight in a N_2_-substituted synthetic medium (termed medium A), pH 7.5, containing 0.2% (w/v) glucose, 0.2% (w/v) yeast extract, 0.04% (w/v) KH_2_PO_4_, 0.1% (w/v) Na_2_HPO_4_·7H_2_O, 0.24% (w/v) NaHCO_3_, 0.03% (w/v) NH_4_Cl, 0.3% (w/v) NaCl, 0.5% (w/v) MgCl_2_, 0.03% (w/v) Na_2_S·9H_2_O, 0.03% (w/v) L-cysteine, 0.1% (v/v) vitamin solution, and 0.1% (v/v) trace element solution using N_2_-substituted, 20-ml rubber-sealed aluminum-cap glass vials (5 ml per vial). The compositions of the vitamin and trace element solutions used in this study are described in Supplementary Tables 1 and 2, respectively. Agar medium A contained 1.5% (w/v) agar in medium A. Strain C5-48^T^ could also grow on GAM agar medium (Nissui Pharmaceutical, Tokyo, Japan) at 37 °C for 24 h in an AnaeroPak container with an anaerobic atmosphere generation system (Mitsubishi Gas Chemical, Tokyo, Japan). C5-48^T^ cells were stored at − 80 °C in broth cultures (medium A) supplemented with 15% (w/v) glycerol.

*Lacrimispora sphenoides* (basonym: *C. sphenoides*) ATCC 19403^T^ [8–10] and *Enterocloster clostridioformis* (basonym: *C. clostridioforme*) JCM 1291^T^ [8, 11] were obtained from the American Type Culture Collection (Manassas, VA, USA) and the Riken BioResource Research Center (Tsukuba, Japan), respectively. The cells of these two type strains were grown in medium A under anaerobic conditions as described above and used for comparative phylogenetic, biochemical, and chemotaxonomic analyses (see below for details).

### Phylogenetic Analysis

Isolation and purification of chromosomal DNA of strain C5-48^T^, *Lacrimispora sphenoides* ATCC 19403^T^, and *Enterocloster clostridioformis* JCM 1291^T^ were performed according to the procedures described by Tamaoka and Komagata [12]. The 16S rRNA gene of strain C5-48^T^ was PCR-amplified from the purified chromosomal DNA using primers 8F and 1492R [13–16]. The PCR products were purified with MicroSpin Columns S-400 HR (Cytiva, Tokyo, Japan) and subjected to sequencing, which was outsourced to GENEWIZ (Saitama, Japan). The nucleotide sequence of 16S rRNA gene of strain C5-48^T^ was submitted to the DDBJ database under accession number LC466003. The nucleotide sequences of genes coding for the β subunit of RNA polymerase (*rpoB*) and heat shock protein 60 (*hsp60*) were obtained from the whole-genome sequence of strain C5-48^T^ (see below). Previously published nucleotide sequences of bacterial 16S rRNA genes were obtained from the EzBioCloud database (http://www.ezbiocloud.net) and the GenBank/EMBL/DDBJ database (http://www.ncbi.nlm.nih.gov/blast). The nucleotide sequences of the *rpoB* and *hsp60* genes were obtained from Integrated Microbial Genomes and Microbiomes Genome (https://img.jgi.doe.gov). Multiple alignments of the sequences of the 16S rRNA, *rpoB*, and *hsp60* genes, calculation of the nucleotide substitution rates [17], construction of phylogenetic trees, and bootstrap analysis for evaluating phylogenetic tree topology [18] were conducted using MEGA software version 11 [19] and CLUSTAL W [20]. Genetic distances were obtained using Kimura’s two-parameter model [17] and clustering was analyzed with the neighbor-joining method [21] using MEGA software version 11 [19], with a bootstrap analysis based on 1000 replicates to estimate the stability of the grouping in each tree.

### Whole-genome Sequence Analysis

To generate genomic DNA libraries, chromosomal DNA prepared as described above was fragmented to approximately 550-bp fragments using an M220 Focused-ultrasonicator (Covaris, Brighton, UK) and fragmented DNA was quantified using a Bioanalyzer 2100 (Agilent, Palo Alto, CA, USA) with the DNA 7500 kit (Agilent). The genomic DNA library was constructed using the TruSeq DNA Library LT kit (Illumina, San Diego, CA, USA) according to the manufacturer’s protocols. Whole-genome sequencing was performed on the Illumina MiSeq system with 2 × 300-bp paired-end reads using a 600-cycle sequencing kit (MiSeq Reagent Kit v3, Illumina). The whole-genome sequence of strain C5-48^T^ was submitted to the DDBJ database with accession numbers BLTJ01000001–BLTJ01000047. Comprehensive average nucleotide identity (ANI) calculations using the genome sequences of strain C5-48^T^ and other prokaryotes available in public databases were performed using the FastANI program [22]. To clarify the genetic relationship between strain C5-48^T^ and the related type strains, digital DNA–DNA hybridization (dDDH) values were calculated using the Genome-to-Genome Distance Calculator 3.0 server (http://ggdc.dsmz.de; formula 2), according to the method described by Meier–Kolthoff et al. [23, 24].

### Phenotypic and Biochemical Analyses

Cell morphology was examined by scanning electron microscopy. In brief, cells during exponential growth phase (see above for growth conditions) were fixed overnight with 2.5% (w/v) glutaraldehyde in 0.05 M sodium cacodylate buffer, pH 7.4, at room temperature, followed by dehydration in a series of graded concentrations of ethanol along with *tert*-butyl alcohol, and were then sputter-coated on a Pt film. Dried cells were then observed using a scanning electron microscope (model S-4800; Hitachi High-Tech, Tokyo, Japan). Motility was checked by phase-contrast microscopy using a model BX40 microscope (Olympus, Tokyo, Japan). Sporulation was examined with cells grown in medium A to stationary phase by phase-contrast microscopy. Gram staining was performed using exponentially-growing cells according to Hucker’s modification [25] with reagents produced by Nacalai Tesque (Kyoto, Japan).

Bacterial growth was monitored for up to 7 days after inoculation by measuring the optical turbidity at 600 nm of cultures in 5 ml of medium A in N_2_-substituted, 20-ml rubber-sealed, aluminum-cap glass vials. An uninoculated vial was used as a control to measure optical turbidity. The bacterial growth temperature range was examined using medium A at 4, 10, 15, 37, 42, and 50 °C (*n* = 3 for each) using a water bath. To determine the pH range for growth, the bacterial cells were grown at 37 °C in medium A, as described above, except that the initial pH of the medium was adjusted with 1 M HCl or 1 M NaOH to pH 5.5, 6.5, 7.5, 8.5, 9.5, or 10.5 (*n* = 3 for each; all pH measurements were performed at room temperature). Phosphate (p*K*_a2_, 6.9), ammonium (p*K*_a_, 9.3), and bicarbonate (p*K*_a2_, 10.3) ions in medium A could serve as buffer components in the examined pH range, although the pH of the medium was not adjusted during bacterial growth. The salt tolerance during bacterial growth was examined at 37 °C, pH 7.5, using medium A except that the NaCl concentration was 2.5%, 4.5%, 6.5%, 8.5%, or 10.5% (w/v; *n* = 3 for each).

Comparisons of the physiological characteristics, including the production of H_2_S, indole, and acetoin, and acid production from carbohydrates, of C5-48^T^, *L. sphenoides* ATCC 19403^T^, and *E. clostridioformis* JCM 1291^T^ under anaerobic conditions were carried out using API 20 E test strips according to the manufacturer’s guidance (bioMérieux Japan, Tokyo, Japan; *n* = 3). The cells were grown as described above and resuspended in water containing 0.85% (w/v) NaCl. The cell suspensions (200 µl) were added to the API 20 E test strip wells, which were placed in an AnaeroPak container with an anaerobic atmosphere generation system (Mitsubishi Gas Chemical) and incubated at 37 °C.

### G + C Contents and Cellular Fatty Acid Profiles

Estimation of the DNA base composition using high-performance liquid chromatography was performed according to the procedures described by Tamaoka and Komagata [12].

The analysis of the cellular fatty acid profiles of strain C5-48^T^, *L. sphenoides* ATCC 19403^T^, and *E. clostridioformis* JCM 1291^T^ were carried out using a Sherlock Microbial Identification System (version 6.0), in which cells were anaerobically grown on GAM agar medium (Nissui Pharmaceutical) at 37 °C for 24 h in an AnaeroPak container with an anaerobic atmosphere generation system.

### Sequence Accession Numbers

The GSDB/DDBJ/EMBL/NCBI accession number for the 16S rRNA gene sequence of strain C5-48^T^ is LC466003. The DDBJ accession numbers of the whole-genome sequence of strain C5-48^T^ are BLTJ01000001–BLTJ01000047.

## Results and Discussion

### Phylogenetic Analysis

**Fig. 1:**
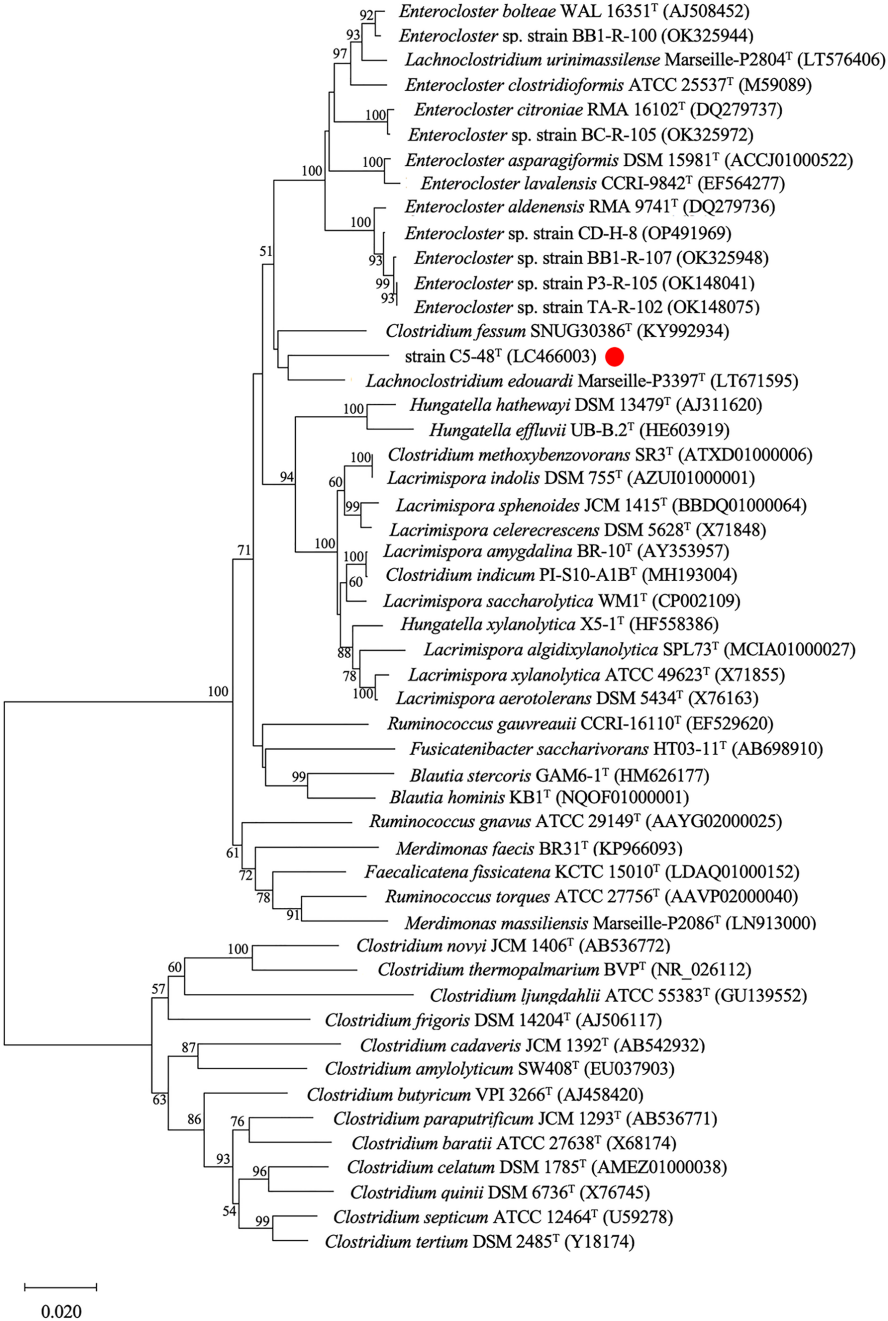
Phylogenetic trees based on 16S rRNA gene sequences showing the relationships between C5-48^T^ (indicated by a *red circle*) and some related taxa. The tree was constructed using the neighbor-joining method, with a bar indicating 0.02 nucleotide substitutions per site. GenBank accession numbers of the nucleotide sequences used are shown in parentheses after strain names. The robustness of the tree was assessed via bootstrapping analysis with 1000 replicates. The numbers indicate bootstrap percentages. The nucleotide sequences of 16S rRNA genes used to generate the phylogenetic trees are also available from Supplementary Data Set 1

**Fig. 2:**
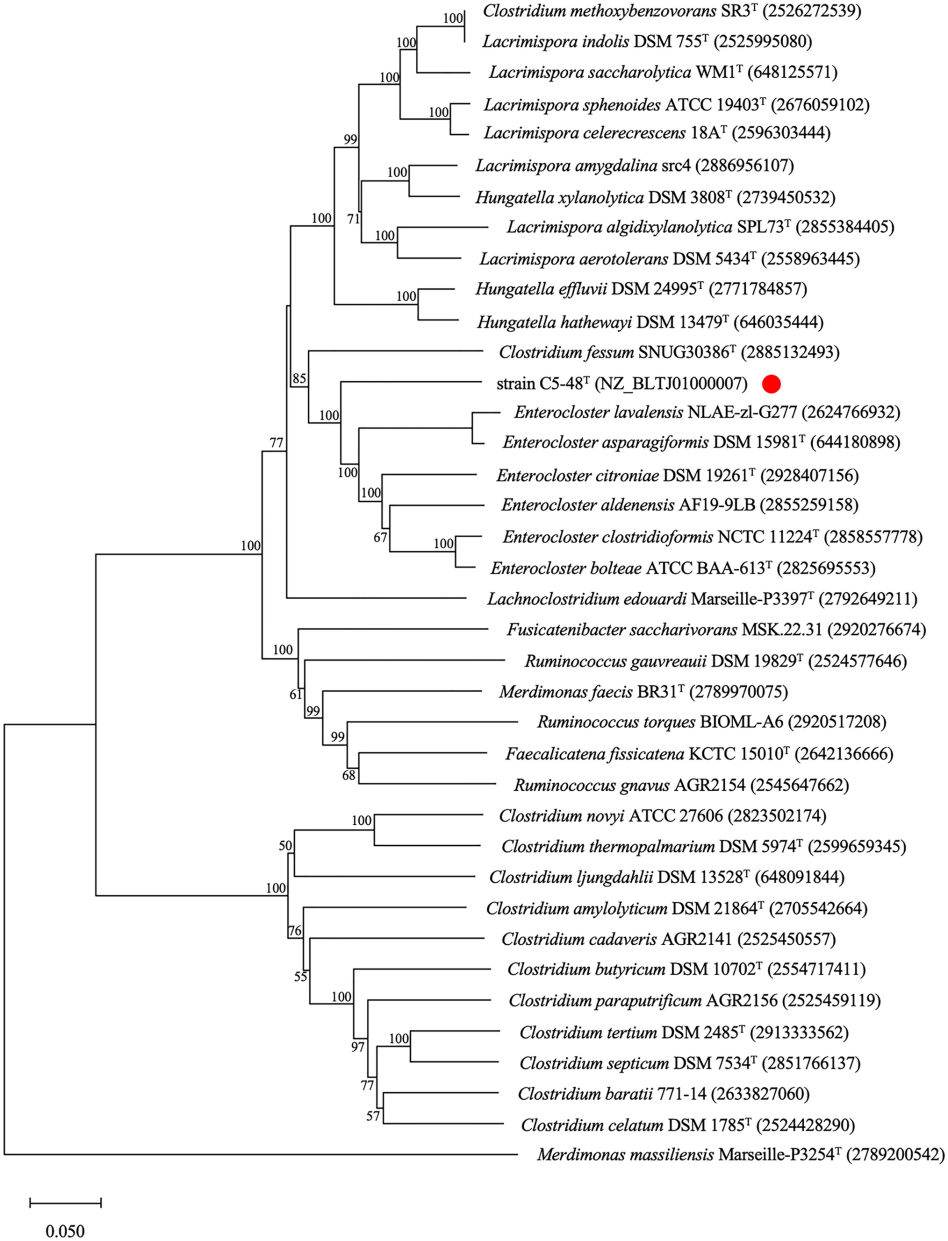
Phylogenetic trees based on *rpoB* gene sequences showing the relationships between C5-48^T^ (indicated by a *red circle*) and some related taxa. The tree was constructed using the neighbor-joining method, with a bar indicating 0.05 nucleotide substitutions per site. GenBank accession numbers (strain C5-48^T^) and Integrated Microbial Genomes and Microbiomes Genome IDs (other strains) of the nucleotide sequences used are shown in parentheses after strain names. The robustness of the tree was assessed via bootstrapping analysis with 1000 replicates. The numbers indicate bootstrap percentages. The nucleotide sequences of *rpoB* genes used to generate the phylogenetic trees are also available from Supplementary Data Set 2

**Fig. 3:**
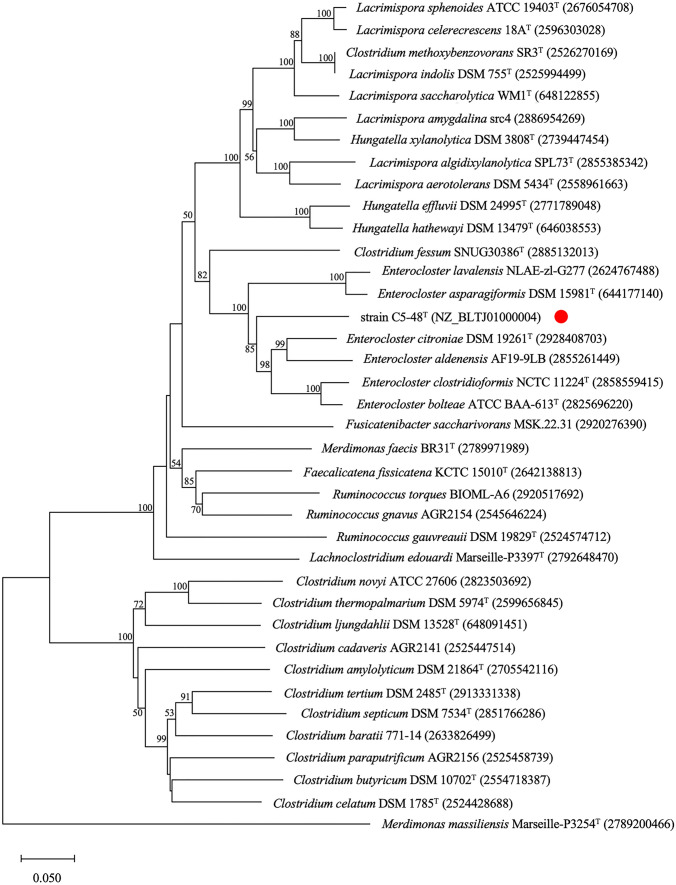
Phylogenetic trees based on *hsp60* gene sequences showing the relationships between C5-48^T^ (indicated by a *red circle*) and some related taxa. The tree was constructed using the neighbor-joining method, with a bar indicating 0.05 nucleotide substitutions per site. GenBank accession numbers (strain C5-48^T^) and Integrated Microbial Genomes and Microbiomes Genome IDs (other strains) are shown in parentheses after strain names. The robustness of the tree was assessed via bootstrapping analysis with 1000 replicates. The numbers indicate bootstrap percentages. The nucleotide sequences of *hsp60* genes used to generate the phylogenetic trees are also available from Supplementary Data Set 3

A 1429-nucleotide stretch of the 16S rRNA gene sequence of strain C5-48^T^ was determined and compared with available 16S rRNA gene sequences. The highest sequence similarity (95.7%) was found with the 16S rRNA gene sequence of *Lachnoclostridium edouardi* Marseille-P3397^T^, which was lower than the cutoff value for the proposal of a novel bacterial species [26, 27]. The 16S rRNA gene sequence of strain C5-48^T^ also showed high similarity to the corresponding sequence of the following type strains: *Clostridium fessum* SNUG30386^T^ (94.7%), *Lacrimispora celerecrescens* DSM 5628^T^ (94.6%), and *Enterocloster asparagiformis* DSM 15981^T^ (94.2%), of which *Lacrimispora* and *Enterocloster* are genera of the Eubacteriales order recently proposed for two previous clostridial groups: the *C. sphenoides* and *C. clostridioforme* groups [8]. Phylogenetic trees were then constructed using the 16S rRNA gene sequences from *Lachnoclostridium*, *Lacrimispora*, *Enterocloster*, *Clostridium*, and related taxa using the neighbor-joining method (Fig. 1). The results showed that C5-48^T^ co-clustered with *C. fessum* SNUG30386^T^ [28] and *L. edouardi* Marseille-P3397^T^ [29], and this cluster was closely related to the *Enterocloster* cluster although these two strains were not previously considered to be strains of *Enterocloster* [8]. To further confirm the phylogenetic position of strain C5-48^T^, we performed phylogenetic analysis based on the nucleotide sequences coding for the β subunit of RNA polymerase (*rpoB*) (see Fig. 2) and heat shock protein 60 (*hsp60*) (see Fig. 3). The results obtained by these analyses consistently supported the inclusion of C5-48^T^ in the genus *Enterocloster* (Figs. 2 and 3)*.* The results of phylogenetic analysis based on these three genes were all consistent with the proposed separation of the genera *Lacrimispora* and *Enterocloster* from the *Clostridium*-related cluster [8] and showed that strain C5-48^T^ was most closely related to the genus *Enterocloster.*

### Whole-genome Sequence Analysis

We also carried out whole-genome sequencing of strain C5-48^T^. The total size of the assembled genome sequence of strain C5-48^T^ was 3.8 Mb, which was similar in size to the genomes of *L. edouardi* Marseille-P3397^T^ and *C. fessum* SNUG 30386^T^ (bacterial strains showing the highest 16S rRNA gene sequence similarity; see above) and smaller than the genomes of many other strains belonging to *Enterocloster* and *Lacrimispora* (Table 1). We then carried out comprehensive dDDH and ANI calculations against prokaryotic genomes available in public databases. The calculated dDDH values of strain C5-48^T^ ranged from 19.4 to 33.5% to related type strains (Table 1), which were lower than the proposed threshold to separate species [30]. The results of ANI calculations revealed that no bacterial species exhibited an estimated ANI value of 95% or higher (Table 1), which is a threshold for species demarcation of prokaryotes [30]. However, some *Enterocloster* and *Lacrimispora* strains were found to have genomic similarities to strain C5-48^T^ with ANI values of 69.4–74.3%, where the values for *Enterocloster* species (72.7–74.3%) were higher than those for *Lacrimispora* species (69.4–70.0%) (Table 1). Thus, although strain C5-48^T^ showed appreciable 16S rRNA sequence similarity to some *Clostridium* or *Lachnoclostridium* species, the strain should most appropriately be assigned to the genus *Enterocloster,* based on its phylogeny derived from the 16S rRNA, *rpoB*, and *hsp60* genes (Figs. 1–3) and its ANI values based on the results of whole-genome sequence analysis.

**Table 1:** Comparisons of the ANI and dDDH values, genome sizes, and G + C contents of C5-48^ T^ and related genome-sequenced strains. Strains are listed in decreasing order of ANI value

Strain	ANI ^*a*^ [%]	dDDH ^*a*^ [%]	Genome size [bp]	G + C [mol%]	Accession No
C5-48^T^	–	–	3,806,033	48.79	GCA_013282095.1
*Enterocloster bolteae* WAL 16351^T^	74.27	26.70	6,570,176	49.10	GCA_002234575.2
*Enterocloster citroniae* MCC335	73.70	23.00	6,509,124	49.49	GCA_018785395.1
*Enterocloster clostridioformis* ATCC 25537^T^	73.44	22.50	5,465,751	48.95	GCA_900113155.1
*Enterocloster asparagiformis* DSM 15981^T^	73.13	22.30	6,353,279	55.51	GCA_025149125.1
*Clostridium fessum* SNUG30386^T^	72.73	33.50	3,277,016	48.29	GCA_003024715.1
*Enterocloster lavalensis* CCRI-9842^T^	72.68	21.30	6,495,707	55.24	GCA_003024655.1
*Lachnoclostridium edouardi* Marseille-P3397^T^	71.18	30.80	3,329,949	42.49	GCA_900240245.1
*Lacrimispora saccharolytica* WM1^T^	69.98	22.00	4,662,871	45.00	GCA_000144625.1
*Lacrimispora celerecrescens* DSM 5628^T^	69.97	23.20	5,272,838	43.89	GCA_002797975.1
*Lacrimispora indolis* DSM 755^T^	69.90	22.80	6,383,701	44.93	GCA_900105615.1
*Lacrimispora sphenoides* JCM 1415^T^	69.90	22.30	5,300,235	43.77	GCA_000421505.1
*Clostridium methoxybenzovorans* SR3^T^	69.76	24.00	7,085,377	44.54	GCA_000421505.1
*Clostridium indicum* PI-S10-A1B^T^	69.67	20.80	5,946,122	42.46	GCA_003432035.1
*Hungatella xylanolytica* X5-1^T^	69.46	22.20	5,629,247	41.85	GCA_002934545.1
*Lacrimispora aerotolerans* DSM 5434^T^	69.44	19.40	4,732,373	42.37	GCA_000687555.1
*Lacrimispora amygdalina* src4	69.40	20.90	5,477,561	41.29	GCA_900205965.1

^a^Values compared with strain C5-48^T^

Estimated annotation of the obtained genome sequence of strain C5-48^T^ revealed 3,479 coding sequences, with the major genes involved in metabolism classified into the following categories: amino acid transport and metabolism (206 genes), nucleotide transport and metabolism (74 genes), carbohydrate transport and metabolism (248 genes), lipid transport and metabolism (38 genes), and inorganic ion transport and metabolism (149 genes, Supplementary Fig. 1).

The genome of strain C5-48^T^ was found to harbor genes encoding enzymes potentially responsible for AcH production from ethanol, such as a putative alcohol dehydrogenase with similarity to AdhE of *Escherichia coli* (UniProt code, P0A9Q7), a putative zinc-type alcohol dehydrogenase with similarity to YjmD of *Bacillus subtilis* (UniProt code, O35045), and a putative catalase with similarity to a catalase of *Lactiplantibacillus plantarum* (basonym: *Lactobacillus plantarum*; UniProt code, P60355). These genes might be related to the observed phenotypic characteristics of this strain as a potential AcH accumulator (see above and ref [6]), although further studies are needed to identify the gene(s) coding for enzyme(s) actually involved in AcH production.

### Phenotypic and Biochemical Characteristics

Cells of strain C5-48^T^ were rod-shaped, 3–4 µm long, and 1 µm in diameter (Supplementary Fig. 2). The cells stained Gram-positive and were non-motile. Endospore production was not observed. Colonies of strain C5-48^T^ that formed after anaerobic growth on medium A agar plates at 37 °C for 72 h were circular, convex, white, and semi-transparent, and their diameter was approximately 1 mm. Strain C5-48^T^ could not grow under aerobic conditions.

Strain C5-48^T^ grew on medium A at pH 5.5–10.5 and at 15–37 °C. The optimum pH and temperature for bacterial growth were 7.5 and 37 °C, respectively (Supplementary Figs. 3 and 4). Strain C5-48^ T^ was slightly salt-tolerant; it could grow in the presence of 10.5% (w/v) NaCl in medium A, although it grew maximally (as determined after 48 h of growth) in the presence of 0.5% (w/v) NaCl (Supplementary Fig. 5).

The biochemical characteristics of C5-48^T^ were examined using API 20 E, and the results were compared with those of *L. sphenoides* ATCC 19403^T^ and *E. clostridioformis* JCM 1291^T^ as summarized in Supplementary Table 3. Strain C5-48^T^ was positive for β-galactosidase activity, arginine dihydrolase activity, tryptophan deaminase activity, H_2_S production, and the Voges–Proskauer reaction (*i.e*., acetoin production), but negative for indole production, lysine decarboxylase, ornithine decarboxylase, urease, and gelatinase. Thus, strain C5-48^T^ was distinct from *L. sphenoides* ATCC 19403^T^ in terms of β-galactosidase and arginine dihydrolase activities and indole production, and from *E. clostridioformis* JCM 1291^T^ in terms of β-galactosidase and arginine dihydrolase activities and H_2_S production. Strain C5-48^T^ could produce acid from L-arabinose, L-rhamnose, glucose, sucrose, melibiose, D-mannitol, D-sorbitol, inositol, and amygdalin, and its acid production profile appeared to be similar to those of *L. sphenoides* ATCC 19403^T^ and *E. clostridioformis* JCM 1291^T^.

### Chemotaxonomic Characteristics

The G + C content of strain C5-48^T^ was determined using HPLC to be 49.4 mol%, while it was 48.8 mol% based on the whole-genome sequence (Table 1), consistent with the G + C contents of other type strains of *Enterocloster* (49–56 mol%), including *E. clostridioformis* ATCC 25537^T^ (49.0 mol%; based on the whole-genome sequence) (Table 1) [8]. For comparison, the G + C content of the type strain of *L. sphenoides* ATCC 19403^T^ was 43.8 mol% (based on the whole-genome sequence).

The fatty acid composition of strain C5-48^T^ is shown in Table 2 compared with those of *L. sphenoides* ATCC 19403^T^ and *E. clostridioformis* JCM 1291^T^. The major cellular fatty acids of strain C5-48^T^ were 16:0, 14:0, and 18:1 *ω*7*c* dimethyl acetal (DMA), and these levels were much higher than those of the other two strains. C5-48^T^ showed lower 18:1 *ω*9*c* and 18:1 *ω*9*c* DMA contents, which were the major fatty acids in the other two strains (Table 2).

**Table 2:** Cellular fatty acid compositions of strain C5-48^T^ and related taxa

Fatty acid	Fatty acid composition (%)		
	**1**	**2**	**3**
10.0	0.1	ND	ND
12:0	0.3	ND	0.3
14:0	22.3	3.0	8.4
16:0	29.9	30.4	34.4
18:0	0.8	0.9	0.7
16:0 ALDE	0.5	0.5	1.6
14:0 DMA	2.0	0.2	1.6
16:0 DMA	2.8	1.8	7.7
18:0 DMA	ND	0.4	0.3
16:1 *ω*7*c*	1.6	5.7	3.4
18:1 *ω*9*c*	0.5	6.6	5.5
16:1 *ω*7*c* DMA	6.3	2.8	3.1
18:1 *ω*7*c* DMA	19.0	14.0	8.3
18:1 *ω*9*c* DMA	1.5	12.6	10.9
Summed feature 1	0.6	0.1	0.6
Summed feature 4	1.6	1.4	1.0
Summed feature 7	ND	2.8	2.0
Summed feature 8	3.3	3.3	1.6
Summed feature 10	6.8	9.7	7.2

Strains: **1**, C5-48^T^; **2**, *Lacrimispora sphenoides* ATCC 19403^T^; **3**, *Enterocloster clostridioformis* JCM 1291^T^. ND, not detected. For strains **1**–**3**, summed features represent groups of two or three fatty acids that could not be separated using the Sherlock Microbial Identification System (ver. 6.0). Summed feature 1 contains C_13: 1_ fatty acid of equivalent chain length (ECL) 12–13, C_14: 0_ aldehyde (ALDE), and/or C_11:1_ 2OH. Summed feature 4 contains an unknown fatty acid of ECL 14.762, C_15: 2_ and/or C_15: 1_ω8*c*. Summed feature 7 contains C_17: 2_ fatty acid of ECL 16.760 and/or C_17: 1_ω8*c*. Summed feature 8 contains C_17: 1_ω8*c* and/or C_17: 2_ fatty acid of ECL 16.801. Summed feature 10 contains C_18: 1_ω7*c* and/or an unknown fatty acid of ECL 17.834

### Taxonomic Conclusion

On the basis of the results of phylogenetic analyses using the sequences of the 16S rRNA, *rpoB*, and *hsp60* genes, comprehensive ANI calculations based on the whole-genome sequence, and cellular fatty acid analysis, C5-48^T^ should be included in the genus *Enterocloster*, which can be clearly distinguished from other closely-related strains. Therefore, we suggest that strain C5-48^T^ represents a novel species of *Enterocloster*, and the name *Enterocloster alcoholdehydrogenati* sp. nov. is proposed.

### Description of *Enterocloster alcoholdehydrogenati* sp. nov.

*Enterocloster alcoholdehydrogenati* (*al.co.hol.de.hydro.ge'na.ti.* N.L. masc. n. *alcohol dehydrogenatus*, aldehyde; N.L. masc. n. *alcoholdehydrogenati*, a bacteria pertaining to aldehyde) is a rod-shaped, non-endospore-forming, anaerobic organism. The cells stain Gram-positive. The rods measure 3–4 × 1 µm. The cell wall mainly comprises 16:0, 14:0, and 18:1 *ω*7*c* DMA fatty acid. The temperature range for growth is 15–37 °C with an optimum of 37 °C. The pH range for growth is 5.5–10.5 with an optimum of 7.5. The strain is slightly salt-tolerant and can grow in the presence of 10.5% (w/v) NaCl in medium A.

The bacterium is positive for β-galactosidase activity, arginine dihydrolase activity, tryptophan deaminase activity, H_2_S production, and the Voges–Proskauer reaction, but negative for indole production, lysine decarboxylase, ornithine decarboxylase, urease, and gelatinase activities. Acid can be produced from L-arabinose, L-rhamnose, glucose, sucrose, melibiose, D-mannitol, D-sorbitol, inositol, and amygdalin. The G + C content of the DNA is 49.4 mol% (based on HPLC analysis) or 48.8% (based on the whole-genome sequence). The type strain is C5-48^T^, which was isolated from the feces of a patient with alcoholism. The type strain has been deposited in the following culture collections: Riken BioResource Research Center (as JCM 33305^T^) and Deutsche Sammlung von Mikroorganismen und Zellkulturen GmbH (as DSM 109474^T^). Strain C5-48^T^ represents a novel species of the genus *Enterocloster*, and the name *Enterocloster alcoholdehydrogenati* sp. nov. is proposed.

## Supplementary Information

Below is the link to the electronic supplementary material.

Supplementary file1 (PDF 1427 KB)

Supplementary file2 (TXT 74 KB)

Supplementary file3 (TXT 143 KB)

Supplementary file4 (TXT 62 KB)

## Abbreviations

ANI: Average nucleotide identity
dDDH: Digital DNA–DNA hybridization
AcH: Acetaldehyde
DMA: Dimethyl acetal
MMC: Minimum mutagenic concentration
