# Supplementary material for: Enterocloster alcoholdehydrogenati sp. nov., a Novel Bacterial Species Isolated from the Feces of a Patient with Alcoholism
Source: Curr Microbiol. 2023 Apr 19;80(5):187. doi: 10.1007/s00284-023-03285-1 (PMC10115706; doi:10.1007/s00284-023-03285-1)
Supplement: Supplementary file 1 — Supplementary file1 (PDF 1427 KB) [file 284_2023_3285_MOESM1_ESM.pdf]

SUPPLEMENTARY INFORMATION

Enterocloster alcoholdehydrogenati sp. nov., a novel bacterial species isolated from the feces of a patient with alcoholism

Daiki Oikawa · Kohei Fukui · Yuichi Aoki · Toshiyuki Waki · Seiji Takahashi · Takefumi Shimoyama · Toru Nakayama

Supplementary Table 1. The formula of trace element solution

EDTA·2Na	0.640	g
$MgSO_4·7H_2O$	6.200	g
$MnSO_4·H_2O$	0.550	g
NaCl	1.000	g
$FeSO_4·7H_2O$	0.100	g
$CoCl_2·6H_2O$	0.170	g
$CaCl_2·2H_2O$	0.130	g
$ZnSO_4·7H_2O$	0.180	g
$CuSO_4$	0.050	g
$AlK(SO_4)_2·12H_2O$	0.018	g
H_3BO_3	0.010	g
$Na_2MoO_4·2H_2O$	0.011	g
$NiCl_2·6H_2O$	0.025	g
Distilled water	1000	ml

Supplementary Table 2. The formula of vitamin solution

Biotin	2.0	mg
Folic acid	2.0	mg
Pyridoxine HCl	10.0	mg
Thiamine HCl	5.0	mg
Riboflavin	5.0	mg
Nicotinic acid	5.0	mg
Calcium D-(+)-pantothenate	5.0	mg
Vitamin B_{12}	0.1	mg
p-Aminobenzoic acid	5.0	mg
DL-α-Lipoic acid	5.0	mg
Distilled water	1000	ml

Supplementary Table 3. Differential characteristics of C5-48^T and related taxa. Strains: **1**, C5-48^T; **2**, *Lacrimispora sphenoides* ATCC 19403^T; **3**, *Enterocloster clostridioformis* JCM 1291^T. +, Positive; −, negative; W, weakly positive.

**Characteristic**	**1**	**2**	**3**
Activity of:			
β-Galactosidase	+	−	+
Gelatinase	−	−	−
Arginine dihydrolase	+	−	−
Lysine decarboxylase	−	−	−
Ornithine decarboxylase	−	−	−
Urease	−	−	−
Tryptophan deaminase	+	+	+
Production of:			
H_2S	+	+	−
Indole	−	+	−
Acetoin	+	+	+
Acid production from:			
L-Arabinose	+	+	+
L-Rhamnose	+	+	+
Glucose	+	+	+
Sucrose	+	W	W
Melibiose	+	+	+
D-Mannitol	+	W	W
D-Sorbitol	+	W	W
Inositol	+	W	W
Amygdalin	+	+	W

a

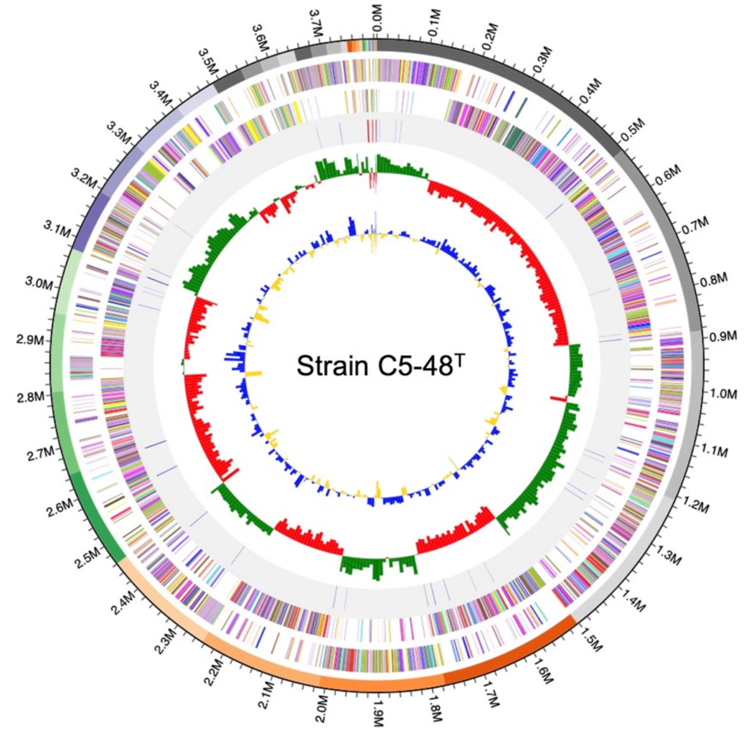

b

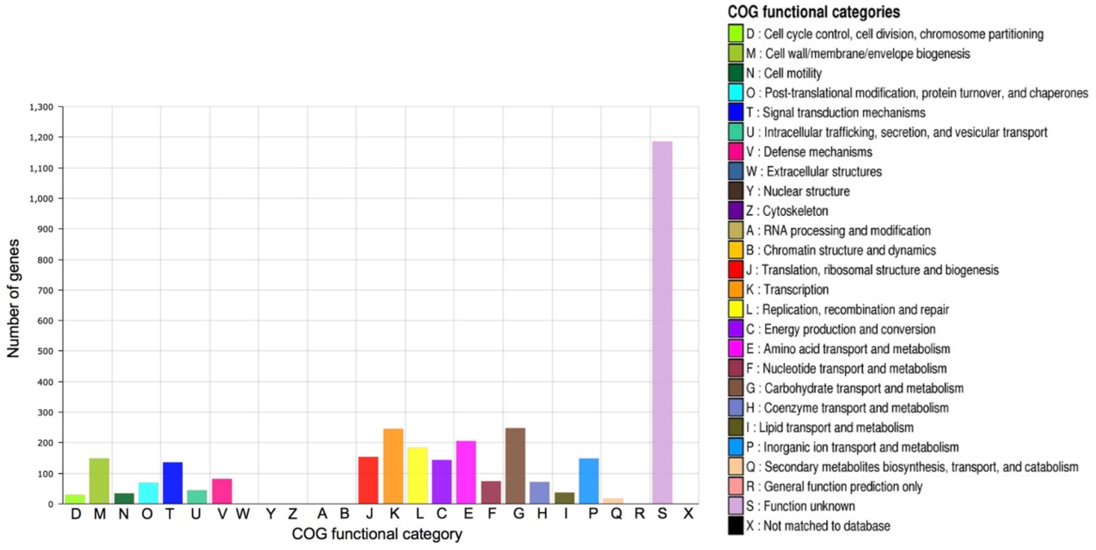

Supplementary Fig. 1. The genome of strain C5-48^T. (a) Genome map of strain C5-48^T. (b) The numbers of clusters of orthologous groups (COG) in the genome of strain C5-48^T. For (a) and (b), COG functional categories are shown in different colors and specified as shown on the right of (b).

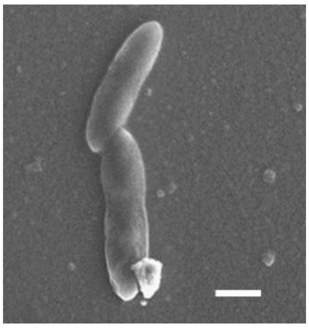

Supplementary Fig. 2. Scanning electron micrograph of strain C5-48^T. Bar = 1 μm. Cells were cultured in medium A for 3 d at 37°C.

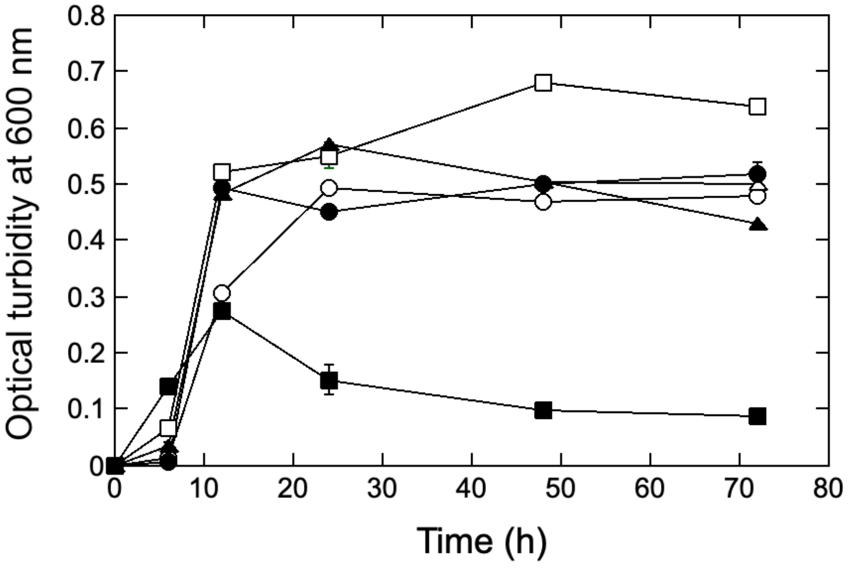

Supplementary Fig. 3. Effect of pH on growth of strain C5-48^{T}. Cells of strain C5-48^{T} were grown at 37°C in medium A, in which the initial pH was adjusted to different values: pH 5.5 (○), 6.5 (△), 7.5 (□), 8.5 (●), 9.5 (▲), or 10.5 (■). Data are presented as the average of three biological replicates (± standard deviation).

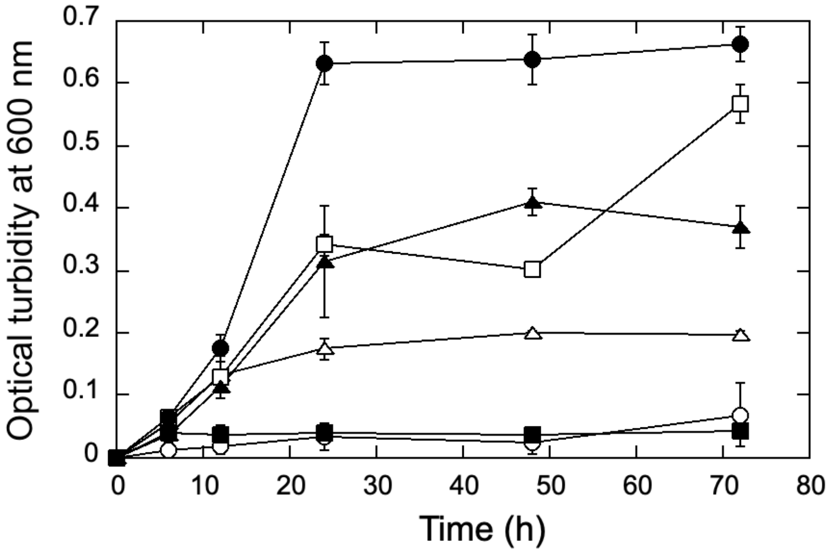

Supplementary Fig. 4. Effect of temperature on growth of strain C5-48^{T}. Cells of strain C5-48^{T} were grown at pH 7.5 in medium A at 4°C (○), 10°C (△), 15°C (□), 37°C (●), 42°C (▲), or 50°C (■). Data are presented as the average of three biological replicates (± standard deviation).

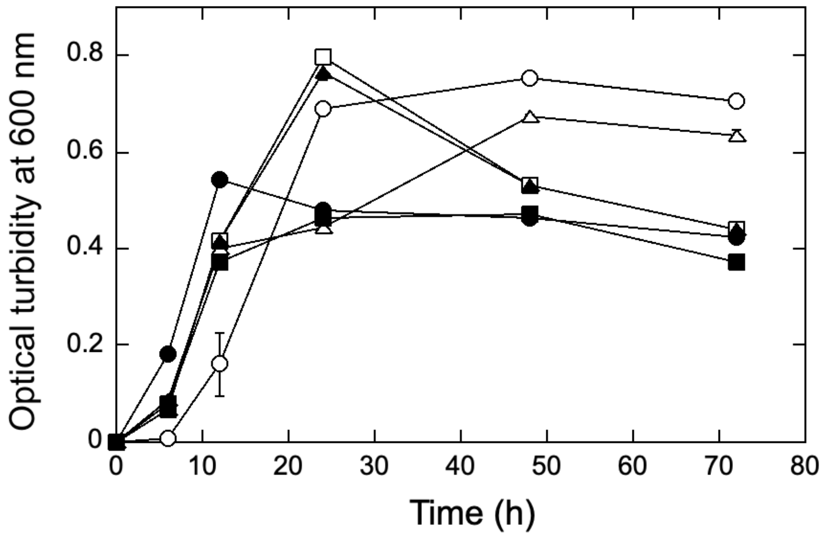

Supplementary Fig. 5. Effect of NaCl concentration on growth of strain C5-48^T. Cells of strain C5-48^T were grown at 37°C in medium A containing the following concentration of NaCl (w/v): 0.5%, ○; 2.5%, △; 4.5%, □; 6.5%, ●; 8.5%, ▲; or 10.5%, ■. Data are presented as the average of three biological replicates (± standard deviation).
